# In Astrocytes the Accumulation of the Immunity-Related GTPases Irga6 and Irgb6 at the Vacuole of *Toxoplasma gondii* Is Dependent on the Parasite Virulence

**DOI:** 10.1155/2013/480231

**Published:** 2013-11-12

**Authors:** Felix P. Lubitz, Daniel Degrandi, Klaus Pfeffer, Anne K. Mausberg

**Affiliations:** ^1^Institute of Medical Microbiology and Hospital Hygiene, Heinrich-Heine-University, Universitaetsstrasse 1, 40225 Duesseldorf, Germany; ^2^Department of Neurology, Heinrich-Heine University, Moorenstrasse 5, 40225 Duesseldorf, Germany

## Abstract

*Toxoplasma gondii* is an obligate intracellular protozoan parasite responsible for a common infection of the central nervous system. Interferon (IFN)**γ** is the key cytokine of host defence against *T. gondii*. However, *T. gondii* strains differ in virulence and *T. gondii* factors determining virulence are still poorly understood. In astrocytes IFN**γ** primarily induces immunity-related GTPases (IRGs), providing a cell-autonomous resistance system. Here, we demonstrate that astrocytes prestimulated with IFN**γ** inhibit the proliferation of various avirulent, but not virulent, *T. gondii* strains. The two analyzed immunity-related GTPases Irga6 and Irgb6 accumulate at the PV only of avirulent *T. gondii* strains, whereas in virulent strains this accumulation is only detectable at very low levels. Both IRG proteins could temporarily be found at the same PV, but did only partially colocalize. Coinfection of avirulent and virulent parasites confirmed that the accumulation of the two analyzed IRGs was a characteristic of the individual PV and not determined by the presence of other strains of *T. gondii* in the same host cell. Thus, in astrocytes the accumulation of Irga6 and Irgb6 significantly differs between avirulent and virulent *T. gondii* strains correlating with the toxoplasmacidal properties suggesting a role for this process in parasite virulence.

## 1. Background

*Toxoplasma gondii* is an obligate intracellular parasite, which is able to infect almost all warm-blooded animals. The virulence of *T. gondii* is strain-dependent. Based on genetic polymorphisms analysis of *T. gondii* isolated from infected patients, the virulence of *T. gondii* was found to be clustered in three classes [1]. This virulence classification was confirmed in mouse infection experiments. While infection with only one parasite of a class I strain is sufficient to kill a mouse, up to 10^5^ parasites are required of a type II or III strain [2]. In an experimental model of a protozoan brain infection, parasite replication is eventually restricted by the immune system. The tachyzoites in the acute stage convert under the immune pressure to bradyzoites initiating the chronic stage of infection [3]. In this silent phase the formed cysts are no longer detected by the immune systems and persist in the CNS—predominantly in astrocytes [4], in skeletal muscles, and cardiac tissue [5]. Nevertheless, occasionally bradyzoite cysts will rupture inducing a rapid recruitment of inflammatory cells [6]. Infection experiments with mice deficient for various genes proved the importance of interferon (IFN)*γ*; IFN*γ*-deficient mice die in the acute phase during the first week of infection [7]. The importance of nonphagocytic cells in the defence against *T. gondii* was clearly demonstrated in bone marrow chimera experiments with IFN*γ*-deficient mice. Thus, for control of *T. gondii* not only the cells of the hematopoietic system which are important but also the cells of nonhematopoietic origin [8]. IFN*γ* induces a whole set of defence mechanisms in classical phagocytotic cells like macrophages. However, astrocytes—the main cell population infected in the brain—are not equipped with most of the defence mechanisms like NO production and indoleamine dioxygenase (IDO) mediated tryptophan degradation [9]. Therefore, up to now, the defence system of cells of nonhematopoietic origin like astrocytes has been unknown. Recently, a new group of IFN*γ* induced p47kDa immunity-related GTPases (IRGs) has been shown to have a major impact on the ability of the host to overcome *T. gondii* infection. Mice deficient for the p47 GTPase Irgm3 (formerly referred to as IGTP) or Irgm1 (LRG-47) succumb within the acute phase of infection [10, 11]. Also for Irga6 (IIGP, IIGP1) a reduced resistance against *Toxoplasma* was demonstrated [12]. In astrocytes the IRGs accumulate at the parasitophorous vacuole (PV) of avirulent ME49 in a GTP-dependent manner which eventually leads to the coordinated membrane vesiculation and destruction of the parasite itself [13]. 

The PV is formed when the parasite invades the host cell. During this process most proteins of the host cell membrane are removed to form a “vesicle” which is not detectable for the immune system [14]. The parasite secretes many proteins in the newly formed PV; a few of them are also transported to the PV membrane and into the host cell cytosol [15]. One group of these important proteins is that of the Rhoptry proteins (ROP). The gene loci determining virulence of *T. gondii* highlighted the ROP2 family, a family of several proteins containing a protein-kinase-like domain [16–18]. Expression of a virulent ROP18 allele in avirulent strains resulted in faster growing parasites and enhanced mortality by 4 to 5 logs in mouse *in vivo* infection experiments [17]. First experiments demonstrated the importance of the pseudokinase ROP5 for the correct ROP18 localisation to the PV [19, 20]. The family member ROP16 on the other hand interacts with host cell signal transduction pathways as it activates regulatory cytokine pathways like IL-4 via STAT6 phosphorylation [21]. 

In the current study, we compare the capacity of astrocytes to combat virulent and avirulent strains of *T. gondii* in terms of parasite replication and kinetics of accumulation of the two important IRGs Irga6 and Irgb6. We further characterized the localization of both IRGs at one individual vacuole and analysed the host cell manipulation of virulent and avirulent strains in coinfection experiments. 

## 2. Methods

### 2.1. *In Vitro* Passage of *T. gondii*

Virulent *T. gondii* strains RH-YFP [22] and BK [23] were maintained in L929 fibroblasts (ATCC, Manassas, USA) and harvested after three days. Harvested parasites in the supernatants were purified from host cell debris by differential centrifugation (5 min at 50 ×g, 15 min at 500 ×g), counted, and used for reinfection. For cultivation of RH-YFP, chloramphenicol (Sigma, St. Louis, USA) was added to maintain selection pressure. Avirulent ME49 [24, 25], NTE [26], and 76K [27] *Toxoplasma* were maintained in HS27 fibroblast (ATCC) and cultivated as described previously [13]. 

### 2.2. Preparation and Cultivation of Astrocytes

Astrocytes were isolated from the brains of neonatal C57BL/6 mice. After decapitation, the brain was prepared, the cortices were isolated, and the meninges were removed. A homogenized cell suspension was prepared as described earlier [13], and 1 × 10^6^ cells/well were seeded in 6-well tissue culture plates in DMEM (10% FCS, 2 mM glutamine, 50 *μ*M 2-mercaptoethanol; Invitrogen, Karlsruhe, Germany). 

### 2.3. Depletion of CD11b^+^ Microglia

After the neonatal cell culture has formed a confluent monolayer (day 10–12), cells were harvested using accutase (Invitrogen). CD11b-positive microglia were depleted using anti-CD11b microbeads based on the manufacturer's protocol (Miltenyi Biotec, Bergisch Gladbach, Germany). The purity of the negative cells containing astrocytes was routinely validated to be more than 98% with a fluorescent-conjugated anti-CD45 antibody (BD Biosciences, Heidelberg, Germany) in a flow cytometer (FACS Canto II, BD Biosciences,). Astrocytes were cultivated for 5–7 days before infection experiments. 

### 2.4. *Toxoplasma* Proliferation Assay

Astrocytes were prestimulated with increasing concentrations of IFN*γ* (0, 10, 100, 1000 U/mL; R&D Systems, Minneapolis, MN, USA) 24 hours prior to infection. Subsequently cells were infected with multiplicity of infection (MOI) of 1 or 0.3 parasites per cell and incubated for 48 hours at 37°C. ^3^H-uracile (Hartmann Analytical, Braunschweig, Germany) was added for the last 24 hours, and *T. gondii* proliferation was measured by detection of the incorporated radioactivity as counts per minute [28] on a Betaplate Counter (LKB Wallac, Helsinki, Finland). Tests were performed as triplicates.

### 2.5. Pulse Infection of Astrocytes with *T. gondii*

Astrocytes on cover slides were prestimulated with IFN*γ* (100 U/mL) 24 h prior to infection. Infection was performed with freshly harvested parasites for 15 minutes at 37°C with MOI of 10 to 30. After 15 minutes, extracellular parasites were removed thoroughly by multiple washing with PBS. Incubation was either stopped or continued with fresh cell medium and IFN*γ* for up to 36 h. Incubation was stopped by fixation with 3% paraformaldehyd (Merck, Darmstadt, Germany) for 20 minutes.

### 2.6. Immunofluorescence Staining

Cells on cover slides were permeabilized and blocked with 0.1% saponin and 1% BSA (Sigma-Aldrich, Munich, Germany) in PBS for 1 h. Both primary and secondary antibodies listed below were incubated for 1 h at room temperature. Between incubation periods, cover slides were thoroughly washed with PBS. Cover slides were fixed on glass slides using fluoromount-G (SBA, Birmingham, UK). 

### 2.7. Immunofluorescence Antibodies

Rabbit anti-Irga6 (1 : 2000, [13]); mouse anti-SAG1 (GII9, 1 : 1000, Innogenetics, Gent, Belgium); goat anti-Irgb6 (1 : 400, Santa Cruz, Heidelberg, Germany) were used as primary antibodies. All corresponding secondary antibodies were purchased from Jackson Laboratories (West Grove, USA) and diluted 1 : 200, except donkey anti-rabbit (1 : 500, Jackson Laboratories) and goat anti-rabbit (1 : 4000, Southern Biotec, USA).

### 2.8. Immunofluorescence Analysis

Fluorescence was analyzed using a microscope Eclipse TE2000S (Nikon, Tokyo, Japan) with the software Lucia Image 4.8.1 (Nikon) and processed with Photoshop (Adobe Systems Inc., San Jose, CA, USA). For Confocal microscopy, an LSM 510 META (Zeiss, Oberkochen, Germany) was used. To avoid crosstalk in the detection of the used fluorophores, multitracking scanning mode was used. Image analyses and processing were performed with the LSM Software (Zeiss).

For quantification of PV in 100 host cells or GTPase recruitment to the PV at least three independent experiments each in duplicates were analyzed for each time point. Parasites were identified either by GRA7-positive parasitophorous vacuoles or by DAPI staining of parasite nuclei. 

## 3. Results 

### 3.1. The Capacity of Astrocytes to Inhibit *T. gondii *Growth Depends on the Parasite Strain

**Figure 1:**
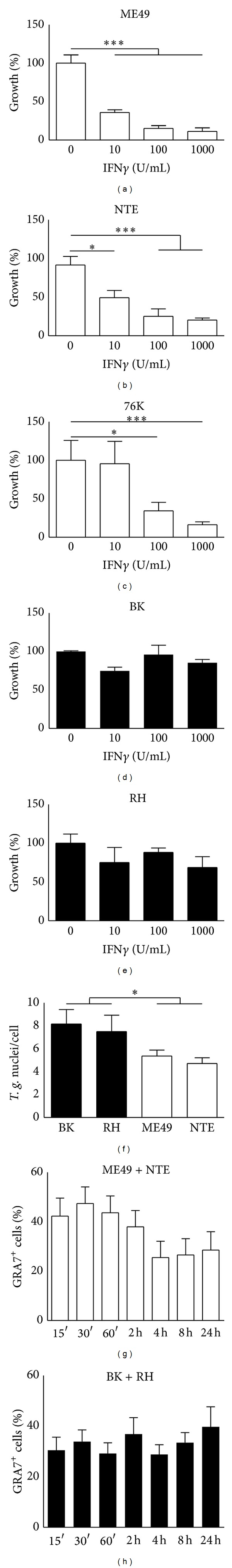
Growth of different *T. gondii* strains in IFN*γ*-stimulated astrocytes. ((a)–(e)) Astrocytes were prestimulated with the indicated IFN*γ* concentrations and infected for 72 h with different *T. gondii* strains (MOI: 1). *T. gondii* growth was measured by incorporation of ^3^H-uracile for the last 24 h of culture using the fact that *T. gondii* is able to incorporate uracile selectively, while in host cells the necessary enzyme is lacking [28]. Uracile incorporation after infection with the different *T. gondii* strains in unstimulated astrocytes was set to 100%, and incorporation in stimulated astrocytes was calculated accordingly. Depicted is mean ± standard error of mean (SEM) of four independent experiments with triplicates (*P < 0.05; ***P < 0.001). Avirulent *T. gondii* strains (white; (a)–(c)) were tested as well as virulent strains (black; (d), (e)). (f) Astrocytes were prestimulated with 100 U/mL IFN*γ* and pulse-infected with the indicated *T. gondii* strains (MOI: 10). After an incubation time of 48 h, infected cells were fixed and tachyzoites per PV were counted by identification of DAPI-labelled *T. gondii* nuclei (mean ± SEM, *P < 0.05). ((g)-(h)) Astrocytes were prestimulated with 100 U/mL IFN*γ* and infected with *T. gondii* (MOI: 10), and after the indicated time points the vacuoles per 100 cells were identified via GRA7 staining. Depicted is mean ± SEM for three independent infection experiments for every strain in duplicates with the averaged results over the avirulent strains (ME49 and NTE, white) and virulent strains (BK and RH, black).

First, we determined the capacity of astrocytes to inhibit proliferation of different *T. gondii* strains by IFN*γ*-dependent mechanisms (Figure 1). In unstimulated astrocytes, growth of avirulent strains was measured by incorporation of ^3^H-uracile in replicating parasites. The replication of the avirulent strains ME49 (Figure 1(a)), NTE (Figure 1(b)), and 76K (Figure 1(c)) was clearly reduced by 80% to 90% in astrocytes prestimulated with IFN*γ* in a dose-dependent manner. In all avirulent strains the analyzed growth inhibition was almost maximal at 100 U/mL IFN*γ*. Similar results were observed when the multiplicity of infection (MOI) was reduced to 0.3 (data are not shown). We, therefore, used 100 U/mL IFN*γ* for further analyses. In strong contrast to avirulent strains, the replication of the virulent *T. gondii* strains BK and RH could not be inhibited by astrocytes (Figures 1(d) and 1(e)). Here, the replication was independent from IFN*γ* stimulation and was not even reduced at highest cytokine concentrations. The reduction of the growth of avirulent strains was confirmed microscopically by counting the number of parasites per infected cell (Figure 1(f)). In prestimulated astrocytes, an average of 8 tachyzoites of the type I strains BK or RH were present per infected astrocyte after 24 h. Infection of IFN*γ*-prestimulated astrocytes with the type II strains ME49 or NTE resulted in a significant reduction to 5 or 6 tachyzoites per cell. The reduced presence of *T. gondii* might have two possible reasons. At the one hand the reduced infection rate could be an effect of the slower replication of the parasite; on the other hand the parasite could also be eliminated by the host cell. To elucidate this, we counted PVs that have been identified via GRA7, a marker for intact PVs. Quantification of intracellular PVs in prestimulated astrocytes over time revealed a reduction in the number of PVs of one-third 4 h post infection (pi) for the summarized data of ME49 and NTE (Figure 1(g)). The number remained stable up to 24 h after infection. In the virulent strains, the number of GRA7^+^ vacuoles was not altered. In contrast, infection rate of cells with the two virulent type I strains (BK and RH) was comparable at 15 min and remained stable to 24 h after infection (Figure 1(h)). Thus, in the avirulent strains, the number of PVs is reduced over time, while in host cells infected with type I virulent strains the number is stable with parasites continuously proliferating within the PV. 

In summary, the results demonstrate that prestimulated astrocytes can inhibit the proliferation of avirulent strains of *T. gondii*. Nevertheless, an inhibition of virulent strains was not observable in astrocytes.

### 3.2. Accumulation Kinetics of Irga6 and Irgb6 at the PV of Different *T. gondii* Strains

**Figure 2:**
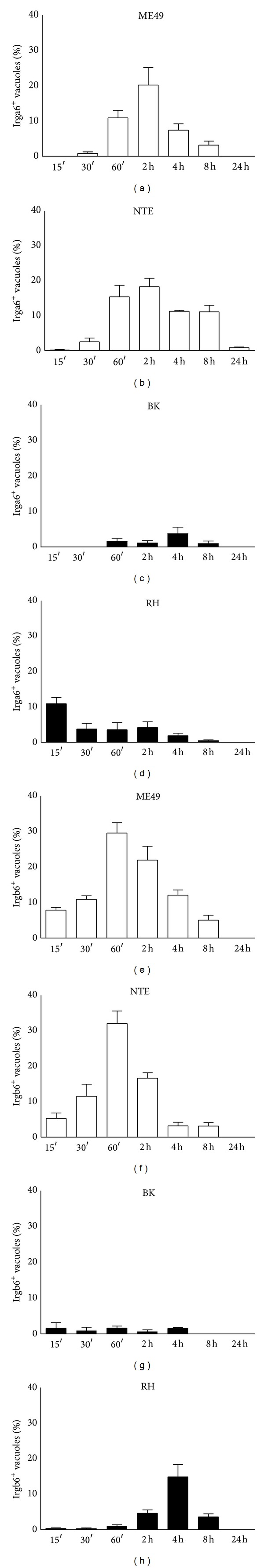
Accumulation of Irga6 and Irgb6 at the PV of different *T. gondii* strains in astrocytes. Astrocytes were prestimulated with 100 U/mL IFN*γ*, pulse-infected with different *T. gondii* strains (MOI: 10) for the indicated time points, and stained for Irga6 ((a)–(d)) and Irgb6 ((e)–(h)). PVs were identified via nucleus staining of tachyzoites with DAPI. ((a)–(d)) Counted Irga6^+^ vacuoles were expressed as percent of all PVs. (a): ME49; (b): NTE; (c): BK; (d): RH. ((e)–(h)) Counted Irgb6^+^ vacuoles were expressed as percent of all PVs. (e): ME49; (f): NTE; (g): BK; (h): RH. The data represent means of five independent infection experiments in duplicates.

To determine the kinetics of Irga6 accumulation, astrocytes were prestimulated, infected with different *T. gondii* strains, and stained for Irga6 at different time points. The parasites were identified by DAPI staining of the characteristic nuclei. In ME49-infected astrocytic monolayers, no Irga6-positive PVs were identified 15 min pi, but the number of Irga6^+^ vacuoles increased stepwise until the maximum was reached at 2 h with 20% of the PVs being positive for Irga6 (Figure 2(a)). After that, the number declined stepwise until almost no positive vacuoles were detectable at 24 h. The second avirulent *T. gondii* strain, NTE, showed a comparable distribution of Irga6-positive vacuoles over time with a maximum at 2 h after infection (Figure 2(b)). In strong contrast to that, in the virulent strains BK and RH the accumulation of the Irga6 protein at the PV was never as high as in avirulent strains (Figures 2(c) and 2(d)). Maximal accumulation at the PVs of BK *T. gondii* was found at 4 h with 5% positive vacuoles (Figure 2(c)). Infection with the type I strain RH demonstrated an unexpected early accumulation at 15 min with 12% of the PVs being positive for Irga6, but already at 30 min this number was reduced below 5% (Figure 2(d)). In analogy to the distribution of the GTPase Irga6, we also investigated Irgb6 distribution (Figures 2(e)–2(h)). Compared to Irga6, the kinetics of Irgb6 accumulation of the PV of avirulent strains (Figures 2(e) and 2(f)) had a related pattern, but Irgb6 accumulation happened earlier with 9% (ME49, E) and 5% (NTE, F) Irgb6^+^ vacuoles being already at 15 min. Also the maximum of the accumulation occurred earlier at 1 h with 32% (ME49) and 33Go (NTE) Irgb6^+^ vacuoles. We could reproduce the described morphological maturation of the IRG localization described by Martens et al. [13] with PVs with a smooth morphology at the early time points (1 h pi), rough vacuoles for the intermediate time points (1 h to 2 h pi), and disrupted vacuoles at the later time points for Irga6 and Irgb6. Quantification of the Irgb6^+^ vacuoles in astrocytes infected with BK parasites showed almost no staining (Figure 2(g)), while in RH-infected cells the maximum was detectable at 4 h pi with 15% vacuoles being positive for Irgb6^+^ (Figure 2(h)). Compared to Irga6, the Irgb6 accumulation was earlier at the PV of avirulent strains. Taken together, both investigated IRGs accumulated time dependently at the PVs of avirulent *T. gondii* strains, while accumulation at PVs of virulent strains was significantly reduced.

### 3.3. Distribution of Irga6 and Irgb6 at the Individual *T. gondii* PV

**Figure 3:**
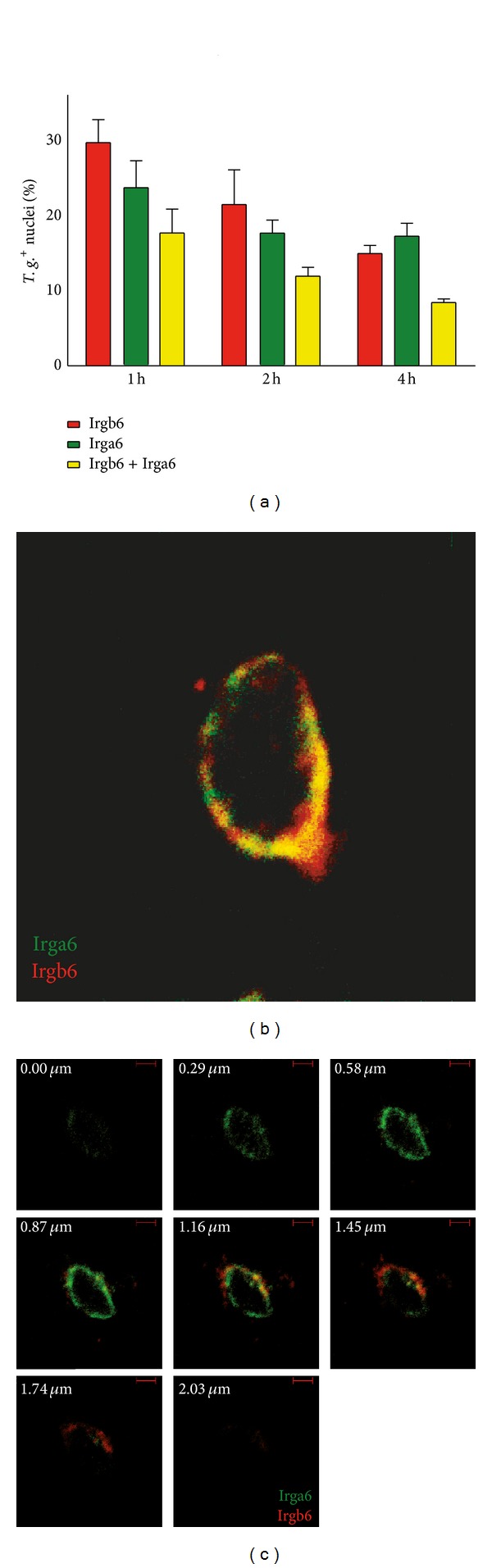
Kinetics and localisation of Irga6 and Irgb6 at the PV of avirulent *T. gondii*. (a) Astrocytes were prestimulated with 100 U/mL IFN*γ*, pulse-infected with ME49 (MOI: 10) for the indicated time points, and stained for Irga6 and Irgb6. PVs were identified via nucleus staining of tachyzoites with DAPI. Quantitative analysis for both IRGs was performed in three independent experiments at the indicated time points mean ± SEM. Red: Irgb6^+^ PV, and green: Irga6^+^ PV, yellow: PV double positive for both analyzed IRGs. ((b), (c)) Astrocytes were prestimulated with IFN*γ* (100 U/mL), pulse-infected with ME49 for 2 h, stained for Irga6 (green) and Irgb6 (red), and analyzed in a confocal microscope. The experiments were repeated three times with comparable results. (b) Representative image of a parasite in a longitudinal section. (c) Representative layered images through a PV with a distance of 0.29 *μ*m between each layer. Note the different distribution of the two IRGs at the PV while scanning through different layers. Bars: 2 *μ*m.

The kinetics of the two IRGs analyzed were slightly shifted. Therefore, we wanted to investigate whether this is a process with one PV being first positive for one and then for the second IRG or some PVs being positive for only a single IRG. In the previous experiments, the kinetics of Irga6 and Irgb6 revealed a peak staining of IRG from 1 h to 4 h. We therefore analyzed these time points and costained ME49-infected prestimulated astrocytes with Irga6 and Irgb6 to analyze the distribution of both IRGs at the PV. The quantification of the GTPases Irga6 and Irgb6 (Figure 3(a)) confirmed that at 1 h almost all Irga6^+^ vacuoles were also positive for Irgb6, while approximately 60 percent of the vacuoles were singly positive for Irgb6. At 2 h and 4 h, the same amount of PV was positive for Irga6 or Irgb6, but only one-third of them were also positive for the other IRG (double positive).  Figure 3(b) depicts a typical cross section of a double-positive parasite. Most of the PVs were double positive indicating colocalisation of both analyzed IRGs, but still some PV areas contained only either one of them. The analysis of layered images (Figure 3(c)) revealed that most of the PVs contained both Irga6 and Irgb6, but the GTPases were partly clustered in single positive areas. Interestingly, at the attached part of the astrocyte the PV showed accumulation of Irga6 while at the medium oriented site Irgb6 is mostly clustered with a ring in the middle where both GTPases are colocalized. This observation was confirmed for most of the analyzed PVs in astrocytes. Although most PVs are double positive for the analyzed time points, the IRG proteins did not colocalize all over the PV.

### 3.4. Accumulation of Irga6 Is Locally Determined by the Individual PV

**Figure 4:**
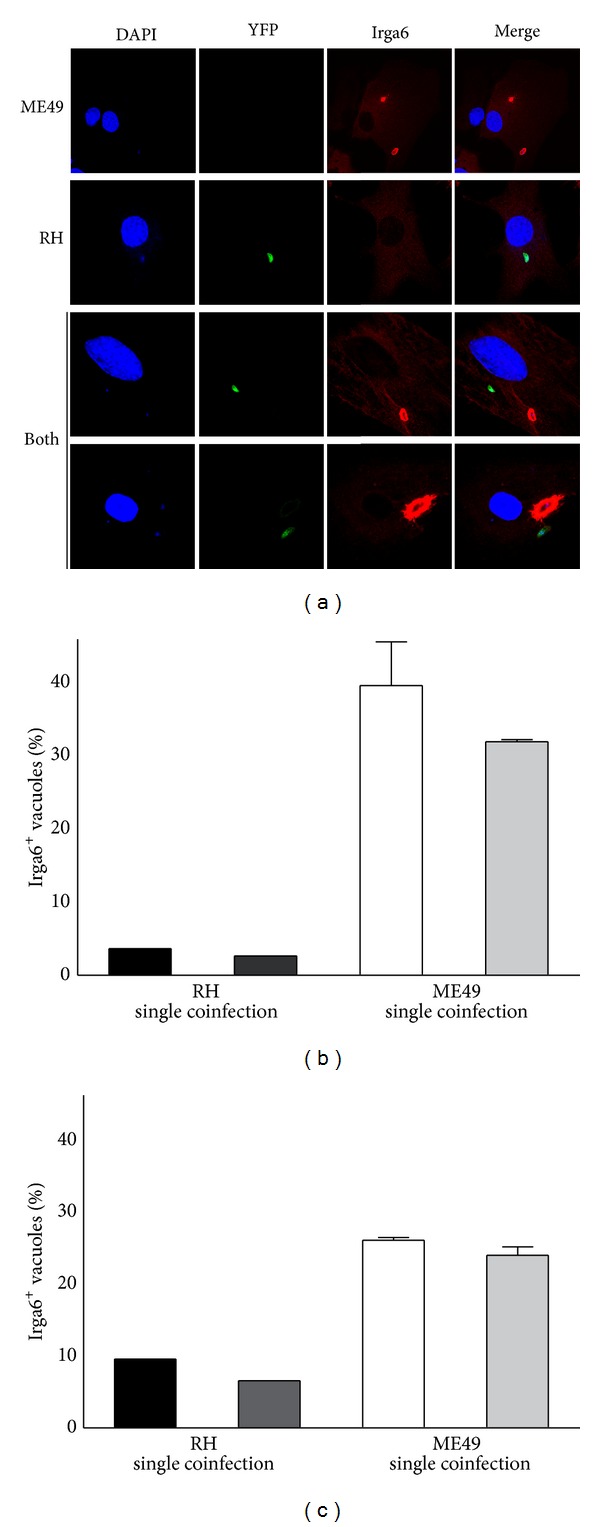
Accumulation of Irga6 in astrocytes coinfected with virulent and avirulent *T. gondii*. Astrocytes were prestimulated with IFN*γ* (100 U/mL) and pulse-infected with either ME49 or RH or simultaneously infected with both strains. (a) Cells were stained for Irga6 (red), costained with DAPI, and analyzed in a confocal microscope. ((b), (c)) After one (b) and two (c) hours Irga6-positive vacuoles are counted in singly infected astrocytes. In comparison coinfected cells were identified containing both strains. Irga6-positive RH containing vacuoles (identified via YFP fluorescence) was distinguished from ME49 containing vacuoles and counted individually.

For the difference in the accumulation of IRGs at the PV of virulent and avirulent strains of *T. gondii,* two possible reasons are conceivable. (1) Virulent and avirulent strains have an altered composition of the PV, and this composition determines the accumulation of IRGs. (2) The parasites affect the host cell capacity to recruit GTPases to any PV. To test these two hypotheses, astrocytes were coinfected with a mixture of ME49 and RH tachyzoites with a comparable infection rate. The recruitment of Irga6 to RH and ME49 containing PV was compared in single infected cells to coinfected cells containing a virulent parasite and an avirulent parasite at the same time (Figure 4). In prestimulated astrocytes infected with ME49, the Irga6 recruitment is demonstrated in a confocal image of DAPI in blue and Irga6 in red. Note the small DAPI-positive parasite nuclei (Figure 4(a)). RH is detectable by the expression of YFP protein in green, but no Irga6 is visible around the virulent RH parasite. In the representative two images of the coinfection, Irga6 is still accumulated at the PV of avirulent ME49, but it is still not detectable at the PV of virulent green RH. To quantify this, Irga6-positive PVs of virulent and avirulent parasites were counted in single infected cells and compared to astrocytes infected with both parasite strains. Regardless whether RH was also present in the same cell, the number of ME49 containing Irga6^+^ vacuoles remained high (between 25–40%) at infection times of one (Figure 4(b)) and two (Figure 4(c)) hours. Corresponding results were obtained when RH containing PVs were counted. Again, the presence of tachyzoites of an avirulent strain had no significant effect on the number of Irga6 accumulation at virulent PVs. Therefore, we concluded that the accumulation of Irga6 at the PV was not affected by the presence of parasites of a different virulence within the same host cell. Thus, the differential IRG accumulation at the PV of virulent and avirulent *T. gondii* appears to be dependent on local factors at the individual PV rather than a general host cell manipulation/interaction by the *T. gondii* parasite. 

## 4. Discussion

Toxoplasmosis in mice is an important model infection to study systemic and intracerebral immune reactions to an intracellular protozoan, since human and murine infections share basic properties. Challenge of mice with low-virulent *T. gondii* cysts induces a disease characterized by an acute and a chronic phase of encephalitis. Astrocytes play a key role in the defence of the infection to *T. gondii* [29]. Even before the intracerebral appearance of *T. gondii* cysts, astrocytes are activated by day 10 pi, most likely as a response to the early invasion of this site by hematogenously spreading tachyzoites [30]. For a long time it was not clear how astrocytes combat the infection against *T. gondii*, given the fact that the common IFN*γ*-induced mechanisms used by classical phagocytotic cells such as macrophages and microglia as NO-and IDO-mediated tryptophan degradation are not detectable in astrocytes [9]. Our data demonstrate that the capacity of astrocytes to inhibit *T. gondii* growth is determined by the virulence of the *T. gondii* strain. In IFN*γ*-prestimulated neonatal astrocyte cultures, the growth inhibition correlates with the increasing IFN*γ* concentrations. In contrast, virulent strains are not inhibited by astrocytes. To examine *T. gondii* strain differences, we analyzed three avirulent (ME49, NTE, and 76K) and two virulent strains (BK and RH) and could observe comparable results within the groups. Astrocytes control the number of tachyzoites per PV of avirulent parasites as well as the percentage of PVs in the host cells indicating a toxoplasmastatical as well as a toxoplasmacidal effect, http://en.wiktionary.org/wiki/toxoplasmacidal.

With the discovery of the IRG gene family—the IFN*γ* responsive p47 GTPases—a key factor determining immune resistance against *T. gondii* was identified [31]. Although the expression of this group of proteins is not conserved in humans, a homologous group of guanylate binding p65 kDa proteins (GBPs) is under discussion to confer this defence mechanism in humans [32, 33]. The mechanism of PV destruction by IRGs is one of the research focuses of the last years [34–36]. Recently, Khaminets et al. discovered the complex interaction of different IRGs to accumulate at the PV with Irgb6 and Irgb10 apparently acting as leading GTPases in the process, while Irga6 accumulation at the PV is a downstream of this process and dependent on the presence of Irgb6 and/or Irgb10 [34]. These findings are in line with our data comparing the kinetics of the two GTPases Irgb6 and Irga6. The kinetic accumulation of Irgb6 was much faster than that of Irga6. Based on our data and the observations of Khaminets et al. it is most likely that once a PV is positive for the leading IRG, the second IRG can be recruited [34]. Eventually the leading IRG is no longer detectable. If this is a maturation process resulting in the dispensability of the leading IRG or if it is substituted by Irgb10 has to be determined in the future. However, it is striking that the defined local distribution of the IRGs revealed a patchy-like clustering of Irga6 and Irgb6 at an individual PV. The area of colocalization was restricted to a small area forming a ring in parallel to the attached astrocyte surface. This ring was observable in different dimensions around most of the surfaces of the PV and seemed to divide the PV in two areas one facing the bottom of the cell/attachment site and the other facing toward the medium site of the astrocyte. It might be speculated that polarisation of the host cell induced by the attached surface on the one hand and the medium site on the other could be a reason for the differences in IRG accumulation. Till now it is not known if this is an astrocyte specific phenomenon or if this is detectable in other polarized cells growing on surfaces (i.e., epithelial cells). Accumulation of the IRG eventually leads to parasite destruction, after the PV membrane peels back the parasite which is exposed to the cytosol [37]. Interestingly, in astrocytes, in murine embryonic fibroblasts, and macrophages virulent *T. gondii* strains are characterized by a reduced loading of Irgb6 on the PV correlating with reduced vacuolar disruption [34, 37, 38]. We could additionally demonstrate the significant growth reduction of avirulent strains in astrocytes, whereas virulent strains with a reduced Irgb6 and Irga6 loading replicated almost unaffectedly. 

The coinfection experiments in this study with avirulent and virulent parasites in the same astrocytic host cell addressed an important question. The differences in accumulation of the two analyzed IRGs at PVs of avirulent and virulent strains could be either explained by local differences in the composition of the individual PVs or by a general interference in host-cell signalling. One example for host cell modulation by *T. gondii* is the protein ROP16, a member of the ROP2 family. Secretion of ROP16 activates STAT3 and STAT6 and reduces proinflammatory cytokines [39]. However, the accumulation of Irg6 at the PV of an avirulent parasite was not altered regardless of the presence of a virulent PV in the same host cell. We therefore conclude that the accumulation of the IRGs is a PV-autonomous feature determined by the virulence of the containing parasite. Since the virulence factors ROP18 and ROP5 are discussed to be responsible for IRG blocking [19, 40], one possible mechanism could be the local targeting of the PV of these two ROP proteins to prevent the IRG accumulation of virulent *T. gondii* strains. Further experiments have to delineate the different compositions of virulent and avirulent PVs.

## 5. Conclusion

In conclusion, we have shown that in astrocytes avirulent and virulent *T. gondii* strains significantly differ in recruitment of the analyzed IRGs to their individual PVs. The amount of IRG recruitment correlates with the inhibitory properties of the astrocyte suggesting a role for this process in parasite virulence. In the accumulation process at the PV, Irga6 and Irgb6 reveal different kinetics and an altered localisation profile. Furthermore, the virulence of the parasite in terms of IRG recruitment seems to be determined by the individual PV of the contained tachyzoites and is not a result of the host-cell manipulation. 

## Abbreviations

IFN: Interferon
IRG: Immunity-related GTPase
MOI: Multiplicity of infection
pi: Post infection
PV: Parasitophorous vacuole
*T. gondii*: *Toxoplasma gondii*.
